# Grain Protein Content Phenotyping in Rice via Hyperspectral Imaging Technology and a Genome-Wide Association Study

**DOI:** 10.34133/plantphenomics.0200

**Published:** 2024-07-08

**Authors:** Hengbiao Zheng, Weijie Tang, Tao Yang, Meng Zhou, Caili Guo, Tao Cheng, Weixing Cao, Yan Zhu, Yunhui Zhang, Xia Yao

**Affiliations:** ^1^ National Engineering and Technology Center for Information Agriculture (NETCIA), MARA Key Laboratory of Crop System Analysis and Decision Making, MOE Engineering Research Center of Smart Agriculture, Jiangsu Key Laboratory for Information Agriculture, Institute of Smart Agriculture, Nanjing Agricultural University, Nanjing, Jiangsu, China.; ^2^ Zhongshan Biological Breeding Laboratory,Nanjing, China.; ^3^ Provincial Key Laboratory of Agrobiology, Institute of Germplasm Resources and Biotechnology, Jiangsu Academy of Agricultural Sciences, Nanjing, Jiangsu, China.

## Abstract

Efficient and accurate acquisition of the rice grain protein content (GPC) is important for selecting high-quality rice varieties, and remote sensing technology is an attractive potential method for this task. However, the majority of multispectral sensors are poor predictors of GPC due to their broad spectral bands. Hyperspectral technology provides a new analytical technology for bridging the gap between phenomics and genomics. However, the small size of typical datasets is a constraint for model construction for estimating GPC, limiting their accuracy and reducing their ability to generalize to a wide range of varieties. In this study, we used hyperspectral data of rice grains from 515 japonica varieties and deep convolution generative adversarial networks (DCGANs) to generate simulated data to improve the model accuracy. Features sensitive to GPC were extracted after applying a continuous wavelet transform (CWT), and the estimated GPC model was constructed by partial least squares regression (PLSR). Finally, a genome-wide association study (GWAS) was applied to the measured and generated datasets to detect GPC loci. The results demonstrated that the simulated GPC values generated after 8,000 epochs were closest to the measured values. The wavelet feature (WF_1743, 2_), obtained from the data with the addition of 200 simulated samples, exhibited the highest GPC estimation accuracy (*R*^2^ = 0.58 and RRMSE = 6.70%). The GWAS analysis showed that the estimated values based on the simulated data detected the same loci as the measured values, including the *OsmtSSB1L* gene related to grain storage protein. This study provides a new technique for the efficient genetic study of phenotypic traits in rice based on hyperspectral technology.

## Introduction

Rice (*Oryza sativa L.*) is a major crop that feeds more than half of the world’s population [1]. With increasing global economic development, there is increasing demand for high-quality rice with high nutritional value. Rice grain protein accounts for approximately 10% of endosperm dry weight and is an important determinant of nutritional quality and taste [2]. Therefore, accurate estimation of rice grain protein content (GPC) is potentially very useful for understanding the genetic basis and breeding of modern high-quality rice varieties.

Since the advent of the 21st century, next-generation sequencing technology has greatly improved the speed and accuracy of genome resequencing and has promoted the development of rice functional genomics and molecular breeding [3]. Genome-wide association study (GWAS) is becoming a powerful tool for bridging the gap between genotyping and phenotyping. Using statistical methods, GWAS can rapidly identify single-nucleotide polymorphism (SNP) loci associated with phenotypic traits and has been widely used for rice genetic dissection [4,5]. However, the low-throughput, costly, and labor-intensive processing of conventional crop phenotyping has created a “phenotype bottleneck,” representing a major challenge for the dissection of target traits [6].

In recent years, the emergence of various optical instruments and analytical techniques has created new opportunities for high-throughput phenotyping. Spectral imaging technology can provide spectral reflectance measurements that are indicative of plant biochemical components and thereby provide nondestructive monitoring through a combination of feature engineering and mathematical modeling [7–13]. Spectral information can also be used to estimate target traits and thus achieve the same genetic analysis efficiency as direct measurement of those traits [10,14,15]. Most previous GPC studies have used visible-near-infrared information from crop canopies [16–18], an approach that does not facilitate monitoring of dry matter absorption features in the shortwave infrared (SWIR) region [19]. Using SWIR measurements of dried seed samples not only avoids the strong masking effects of water absorption but also allows the exploration of causal relationships between chemical components and absorption characteristics, thus laying the framework for the construction of a robust estimated GPC model [20].

Hyperspectral reflectance measurements are typically made in multiple narrow wavelength regions, termed spectral bands. Because these spectral bands are generally much narrower than the plant spectral absorption features, spectral reflectance data tend to be characterized by redundant information, which increases the computational burden and reduces the stability of models that use the data. To address this problem, feature reduction methods that reduce the number of predictor variables can be used. The types of feature reduction approaches include feature selection methods, such as partial least squares regression (PLSR), successive projection algorithms and genetic algorithms, and feature extraction methods, such as principal components analysis (PCA), linear discriminant analysis (LDA), and continuous wavelet transform (CWT). CWT, an effective method for quantifying vegetation physiochemical properties from reflectance spectra, has been successfully applied to extract spectral features in hyperspectral remote sensing [21–24]. CWT decomposes raw spectral data into wavelet features (WFs) of different magnitudes and scales to facilitate the identification of both overall and subtle features and provides information directly comparable to the original spectral bands [25]. Nevertheless, CWT has only rarely been used for extracting features sensitive to seed proteins.

The size of the dataset used in building classification or regression models is an important consideration. For most models, including k-nearest neighbors, support vector machines, and convolutional neural networks, increasing the number of training samples improves performance [26] until a plateau is reached [27]. However, in practice, datasets are often limited and unbalanced in terms of the proportion of samples in different classes because collecting data is usually limited by labor and material resources. In particular, it is difficult to obtain large research datasets that include a wide variety of rice germplasm data. Using limited datasets constrains the subsequent data analysis and increases uncertainty in the interpretation.

Data augmentation offers an effective method for addressing this problem by increasing the number of training samples. Many studies have shown that data augmentation can reduce overfitting and improve model performance [28–30]. Generative adversarial networks (GANs) have been shown to generate simulated data that are realistic to human evaluators. Through the use of a novel loss function that distinguishes between simulated and real data, a GAN can be extended from classification to regression problems [31]. In combination with real data, deep convolution generative adversarial networks (DCGANs) have been found to be useful for generating simulated corn kernel spectral data and associated oil content information, improving the performance of regression models [32]. However, in past studies, the data for training GANs have often originated from relatively homogeneous samples, with few varieties. The numerous varieties of rice result in heterogeneity that may increase noise in the simulated data. Thus, the feasibility of using a GAN for data augmentation for rice varieties is unclear. Furthermore, most previous studies have focused only on monitoring and predicting target traits, ignoring genetic dissection results of estimated values in breeding.

Therefore, this study aimed to solve the problem of limited training datasets by using a DCGAN to construct a high-generalization GPC model applicable to a wide range of rice varieties and to explore the model’s gene dissection potential. The main research objectives are (a) to use a DCGAN to generate simulated data and to extract WFs associated with GPC, (b) to construct rice GPC estimation models and to evaluate what effects (if any) the simulated dataset had on the regression model, and (c) to identify GPC-related genes through GWAS analysis from measured and estimated GPC values.

### Study site, data collection, and data preprocessing

#### Field data collection

Two field experiments were conducted in 2020 and 2021 in Nanjing, Jiangsu Province, China (118°46′E, 32°02′N) (Fig. S1). Experiment 1 was performed at the Jiangsu Academy of Agricultural Sciences experimental farm. A total of 230 varieties of japonica rice from the Taihu Basin were planted in 2020 and again in 2021 (Table 1), with each variety planted in a separate plot approximately 0.6 m × 1.5 m in size. N fertilizer was applied at 2 stages: 50% as basal fertilizer before transplanting and 50% at the jointing stage. The rice was harvested at full maturity. Experiment 2 was located at the rice breeding base of Nanjing Agricultural University. A total of 190 varieties were planted in 2020, and 320 varieties were planted in 2021. Each variety was planted in a separate plot of approximately 0.9 m × 1.5 m. The rest of the cultivation practices were the same as those for experiment 1. Over the 2 years and the 2 experiments, a total of 970 varieties were planted, but only 514 survived to harvest due to disease or other problems (Table S1).

**Table 1.** Training sets based on different numbers of measured (i.e., real) and simulated samples

Sample set	Number of samples	
	Measured	Simulated
Validation set	238	0
Training set 1	50	0
Training set 2	150	0
Training set 3	276	0
Training set 4	276	50
Training set 5	276	100
Training set 6	276	200
Training set 7	276	300
Training set 8	276	500
Training set 9	276	700
Training set 10	276	997

#### Hyperspectral image acquisition and correction

After the dry panicles were threshed and ground into polished grains, approximately 1,000 grains of each variety were randomly selected. The samples were laid flat without a cover in 2 containers, each with a radius of 5 cm, and previously wrapped in black cardboard with a reflectance of approximately 10%.

A push-broom SWIR system (Image-λ-N25E-HS, Jiangsu Dualix Spectral Image Technology Co. Ltd., China) loaded on a conveyor was used to collect hyperspectral data from each container of grain samples (Fig. S2). The hyperspectral system provides 256 spectral bands over a wavelength range of 980 to 2,500 nm, with a spectral resolution of 6 nm. The camera exposure time was set to 11 ms, the distance from the lens to the sample was 29.5 cm, and the conveyor motion speed was 0.73 cm·s^−1^. To avoid interference from external light, data acquisition was carried out in a dark room.

The hyperspectral images were analyzed using the Python programming environment (version 3.8, available at http://www.python.org). The following operations were carried out to obtain grain SWIR spectra for each rice variety from the image hypercubes:

1. Reflectance conversion. The digital number of each pixel was converted to reflectance using Eq. 1, and the absorbance values were then calculated as log_10_(1/*R*) [33], where *R* is the reflectance:

$$R_{t}=\frac{{\mathrm{DN}}_{o}-{\mathrm{DN}}_{d}}{{\mathrm{DN}}_{w}-{\mathrm{DN}}_{d}}\times R_{w}$$ (1)

where *R*_t_ is the reflectance of the target; *DN*_o_ is the DN value of the original hyperspectral image; *DN*_d_ is the DN value of the dark current; *DN*_w_ is the DN value of the white Teflon standard; and *R*_w_ is the reflectance of the white Teflon standard.

2. Image masking. Pixels with a difference of more than 0.5 between the absorbance values at 1,300 and 1,450 nm were set as the foreground, and the remaining pixels were masked.

3. Noise reduction. Pixels with a value greater than ±2 SDs of the mean spectrum were removed.

4. Reflectance averaging. The remaining pixels after masking and noise reduction were averaged for subsequent statistical analysis.

#### GPC measurement

After spectral data collection, the grains were milled into rice flour, and the samples were preprocessed by HClO_4_-H_2_SO_4_ digestion. Then, the total N in the grain (%) was determined using the semimicro Kjeldahl method [34] and a SEAL AutoAnalyzer 3 HR (SEAL Analytical Ltd., Germany). GPC was determined to be 5.82 times greater than the total N in the grain [20,35].

#### Genome-wide molecular data

SNP loci data for 230 of the 276 rice varieties harvested in experiment 2 were obtained from the Institute of Germplasm Resources and Biotechnology, Jiangsu Academy of Agricultural Sciences (unpublished data). The Illumina HiSeq platform was used for the resequencing procedure. On average, 57.41 million mapped reads per sample were produced, and the sequencing depth was approximately 21.7X. The sequence of the japonica variety Nipponbare was used for reference. The fastp (https://github.com/OpenGene/fastp), bwa (http://sourceforge.net/projects/biobwa), and GATK4 (https://github.com/broadinstitute/gatk) software programs were used for resequencing data quality control, mapping, and variation calling. Admixture (http://dalexander.github.io/admixture) and TASSEL5 (https://tassel.bitbucket.io/) software were used to increase the model parameters Q and K.

Plink software (version 1.9, https://www.cog-genomics.org/plink/1.9/general_usage) was used to perform quality control analysis on the SNP sites. To decrease the number of false-positive sites, the following screening conditions were used: SNP loci with a minor allele frequency > 0.05 and a call rate > 90% were retained. This resulted in 653,465 polymorphic and high-quality SNP markers being retained for subsequent analysis.

## Methods

### GPC estimation and GWAS workflow

**Fig. 1.**
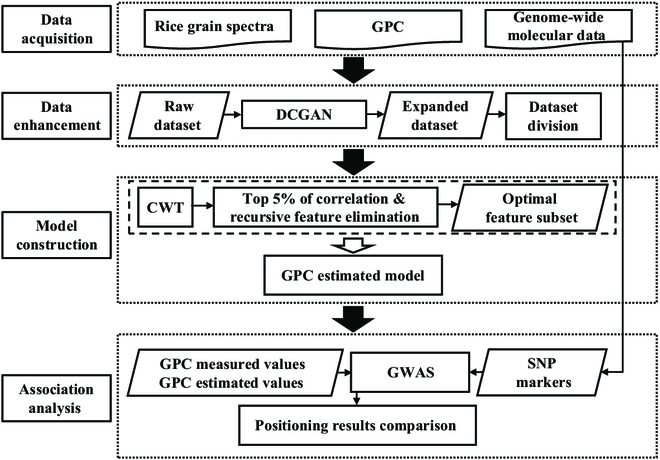
Workflow of GPC estimation and GWAS.

The data analysis workflow is shown in Fig. 1. First, the raw dataset (rice grain spectra + GPC) was used to train the DCGAN model, and a large, simulated dataset was generated to construct an expanded training dataset. Then, CWT was used to generate features. Redundant features were eliminated using correlation analysis and recursive feature elimination, and the resulting optimal feature subset was used to construct a GPC estimation model. Finally, the results of the GWAS for the measured and estimated GPC values were compared.

### Data augmentation using the DCGAN

#### Construction of the DCGAN

We used the DCGAN to address the problem of poor parameter tuning and thereby improve the training speed and stability of our predictive models. A GAN is a deep learning model that learns the original distribution of data and generates instances similar to the original data through continuous training [36]. GANs consist of 2 main components, a generative model (G) and a discriminative model (D), which compete with each other to produce progressively better outputs. The DCGAN improves upon the GAN through modifications to the network structure [37]. Specifically, convolutional neural networks (CNNs) are used in both G and D, and the CNN structure is also improved. The simplicity of the DCGAN structure makes it easy to use with one-dimensional spectral data.

In our implementation, a DCGAN was used to simultaneously generate simulated spectra and the associated GPC data using the following procedures:

1. We defined the spectral vector for each variety as *S_n_* = (*S*_1_, *S*_2_, …, *S_n_*) and a scalar, *g*, for the GPC (*n* is the number of bands and *Si* is the absorbance value).

2. A maximum-minimum normalization of *S* was performed to accelerate the convergence of the DCGAN model, and *g* is divided by 10. *Si*∈[0, 1].

3. Since 1,510 nm is a protein absorption band, a new vector *V*_*n*+1_ = (*S*_1_, *S*_2_, … *g*…, *S_n_*) was formed after inserting GPC into the 1,510-nm band of the corresponding varieties.

4. The DCGAN model was trained using the combined data and used to generate simulated data.

**Fig. 2.**
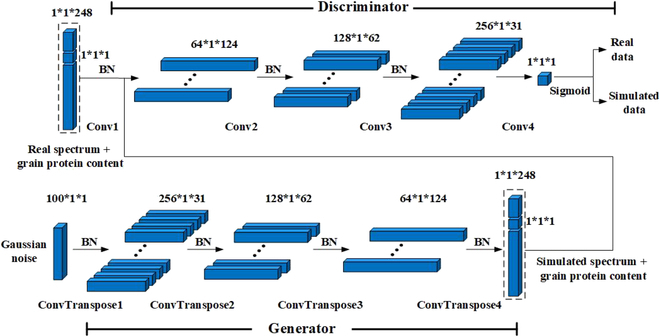
Diagram of the DCGAN structure, including the discriminator, D, and generator, G.

Figure 2 shows the structure of the DCGAN based on a one-dimensional CNN. The input to G is 1 × 1 × 100 Gaussian noise, and the output is 1 × 1 × 248 simulated data points. D consists of a convolutional layer, batch normalization (BN) layers, and a LeakyReLU activation function. The input to D is 1 × 1 × 248 one-dimensional data (including 1 × 1 × 247 spectral data and 1 × 1 × 1 GPC since 8 spectral bands influenced by noise are deleted). A sigmoid activation function was applied prior to predicting whether the input was real or simulated data. The estimation model was trained to generate spectra and GPC-simulated data with a batch size of 8 and a maximum of 10,000 epochs.

#### Dataset division and statistical analysis

The raw training dataset for this study was the output from experiment 2, including data from 2020 and 2021, representing a total of 276 samples. The validation set comprised 238 samples from experiment 1 in 2020 and 2021. To evaluate the effectiveness of DCGAN data augmentation, we followed the procedures of Zhang et al. [32], using training sets of varying sample sizes to train regression models and a single, consistent dataset for validation. As summarized in Table 1, training sets 1 and 2 were generated by randomly sampling 50 and 150 samples, respectively, from the real dataset. The entire real dataset (i.e., 276 samples) formed training dataset 3. Training Sets 4 to 10 were formed from the entire dataset combined with between 50 and 997 simulated samples. For each training set, the random sample selection and model training process was repeated 10 times, and the average of the evaluation metrics was used to characterize the model.

As the number of iterations increased, the parameters of the DCGAN were constantly updated. We therefore used a boxplot to summarize the similarity between the measured and simulated datasets as the number of iterations increased.

### Feature extraction and model construction

#### Continuous wavelet transform

Wavelet analysis can be used to perform multiscale analysis of information in 1-dimensional signals or 2-dimensional images [38]. In particular, wavelets have recently been applied in hyperspectral remote sensing as a spectral analysis tool for extracting meaningful spectral information from spectral curves [20,21,39]. In CWT, wavelet coefficients at different scales are obtained by scaling and translating the mother wavelet function and then performing convolution operations on the spectral data. The transformation formulas are as follows:

$$\psi_{\;a,b}\left(\lambda \right)=\frac{1}{\sqrt{a}}\psi \left(\frac{\lambda -b}{a}\right)$$ (2)

$$W_{f}\left(a,b\right)=\langle f,\psi_{\;a,b}\rangle =\int_{-\infty }^{+\infty }\;f\left(\lambda \right)\psi_{\;a,b}\left(\lambda \right)\mathrm{d\lambda}$$ (3)

where *ψ*(*λ*) is the wavefunction; *λ* is the spectral band; *ψ*_*a*,*b*_(*λ*) is the mother wavelet function after scaling and translation, with *a* as the scaling factor, also known as the scale, and *b* as the translation factor; and *W_f_*(*a*, *b*) is the wavelet coefficient, also called the WF. A Gaussian second-order derivative was used as the mother wavelet function in this study. The scales used were 2, 3, 4, and 5.

#### Model construction

Four representative algorithms were selected for regression model construction: PLSR, support vector regression (SVMR), random forest regression (RFR), and Bayesian linear regression (BLR). During model construction, the hyperparameters of each model were determined using fivefold cross-validation and a grid search, and the mean squared error (MSE) was used as the evaluation metric.

To avoid instability in the model estimation and bloating of model input parameters caused by strong correlation between the many input features, we selected 5% of the bands with the highest correlation with GPC values. Recursive feature elimination was then employed to further streamline the features, and the optimal feature combination was determined. Finally, the GPC estimated models were constructed using the full-band WFs and the sensitive features from the different training sets.

#### Accuracy assessment

Independent validation was conducted to evaluate the transferability and predictability of the GPC estimation models. The models were evaluated using the coefficient of determination (*R*^2^), root mean square error (RMSE), and relative root mean square error (RRMSE).

$$R^{2}=1-\frac{\sum_{i=1}^{N}\;{\left(y_{i}-{\hat{y}}_{i}\right)}^{2}}{\sum_{i=1}^{N}\;{\left(y_{i}-\bar{Y}\right)}^{2}}$$ (4)

$$\text{RMSE}=\sqrt{\frac{1}{N}\sum_{i=1}^{N}{\left(y_{i}-{\hat{y}}_{i}\right)}^{2}}$$ (5)

$$\text{RRMSE}=\frac{\text{RMSE}}{\bar{Y}}\times 100\%$$ (6)

where *N* is the total sample size, *y_i_* is the measured value, ${\hat{y}}_{i}$ is the average of the measured values, and $\bar{Y}$ is the estimated value. For model validation, *R*^2^ differs from the squared correlation coefficient and is a better measure of data fitting to the 1:1 line. Following Li et al. [40], negative *R*^2^ values were set to zero to avoid confusion.

### Genome-wide association study

GWAS based on linkage disequilibrium (LD) uses statistical tools to identify associations between target traits and genetic variants. With the help of a high-frequency genetic map consisting of millions of SNP markers, it is possible to screen for SNPs that may be associated with variations of interest. To remove the influence of population structure on association analysis, kinship and population structure matrices can be added to the model [41]. Therefore, the association analysis model chosen for this study was the mixed linear model (MLM) with the following equation:

Y=Xα+Qβ+Kμ+e (7)

where *Q* represents the population structure matrices, *K* represents the kinship matrices, *X* is the genotype value, and *Y* is the phenotype value.

The number of varieties included in the GWAS was 158 since 72 varieties failed to mature due to lodging and disease. The threshold for significance was assessed using the Bonferroni method, i.e., *P* = 0.01/*n*, where *n* indicates the number of valid SNPs. Only the associated SNPs exceeding the significance threshold were considered. To avoid overcorrection, the significance threshold, log_10_(*P*), in this paper was set to 4 [42].

The LD decay distance of the natural variant population of rice was approximately 200 kb [43]; therefore, a physical distance between any 2 significant SNPs less than 200 kb was considered a locus. The SNP with the greatest significance in the interval containing more than 3 consecutive significant SNPs was selected as the lead SNP.

## Results

### Estimation of rice GPC based on the DCGAN

#### Hyperspectral data analysis

**Fig. 3.**
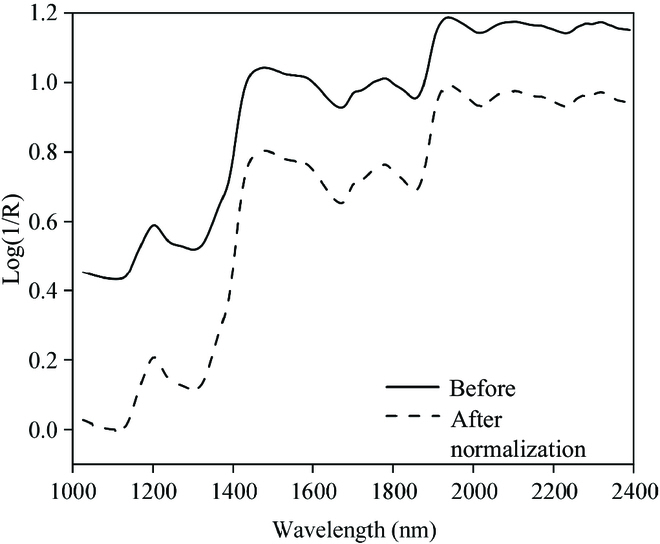
Raw and normalized average absorbance spectral curves of grains.

Figure 3 shows the rice grain raw and normalized average absorbance spectral curves obtained during preprocessing. Due to the strong absorption of dry matter in the SWIR region, numerous absorption features can be identified, especially after normalization, with the most distinct peaks near 1,200, 1,450, 1,800, and 1,950 nm. The absorption peak near 1,200 nm is mainly from the bending and first-order overtoning of O–H bonds in cellulose, starch, and lignin [19]. The peak near 1,450 nm is mainly from the stretching and first-order overtoning of O–H bonds in starch and sugars and the stretching of C–H bonds in lignin. The peak near 1,800 nm is mainly from the stretching and first-order overtoning of C–H bonds in starch and sugar; the peak near 1,950 nm is mainly influenced by lignin, protein, and starch.

#### Analysis of the similarity of the simulated data and measured data

**Fig. 4.**
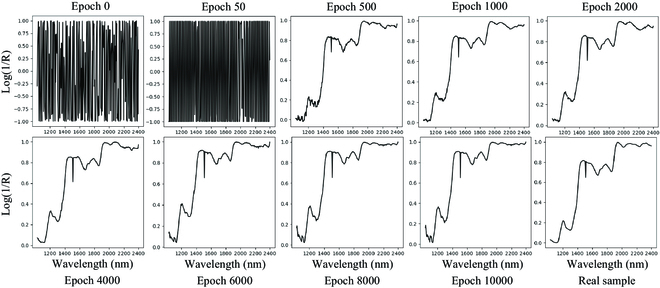
Comparison of the measured sample and the simulated samples after various epochs.

Figure 4 shows the comparison between the measured data and the simulated data generated by the DCGAN after an increasing number of iterations. At epoch 0, the generated data are simply random noise. After epoch 500, the generated sample already has an overall prototype of the measured sample, and the peaks and valleys of the spectral curve are generally consistent with those of the measured sample. However, at this stage, the spectrum still contains considerable noise, especially at the edges of the curve. As the number of epochs further increases, the overall shape of the simulated spectral curve remains unchanged, but the curve tends to become smoother. By epoch 4000 and for subsequent epochs, the generated spectral curve is very close to that of the measured sample, and it is only through statistical assessment that the similarity can be quantified.

**Fig. 5.**
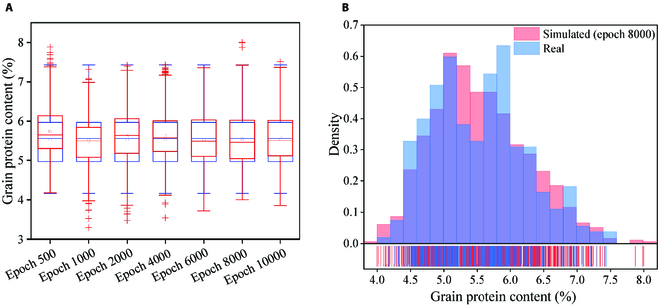
Box plot of measured and simulated GPC data generated after increasing numbers of iterations (A) and frequency histogram of measured and simulated GPC after 8,000 iterations (B).

Figure 5A shows a box plot of the measured and simulated GPC data generated after various numbers of iterations. As the number of iterations increases, the distribution of the simulated data gradually approaches that of the measured data. When the number of epochs reaches 8,000, the 2 distributions are the most similar. At this point, the 2 quartiles of the simulated data and the maximum are almost consistent with the measured data, and there is only a slight difference in the mean and the minimum values. However, there is a notably large difference in the frequency of samples at approximately GPC = 5.4% (Fig. 5B). When the number of epochs increases beyond 8,000, the similarity between the real and simulated data starts to decrease. Therefore, for generating the 1,000 samples for the data augmentation and model construction, we set the number of epochs to 8,000.

After removing the outliers, all the statistics between the measured and simulated datasets are very close, except that the minimum value of the simulated dataset is notably smaller than that of the measured sample (Table S2). In addition, the validation set has a greater mean and a lower coefficient of variation (CV) than the training set.

#### Regression model selection

To determine the most suitable regression algorithm, we compared the accuracies of the 4 different GPC estimation models constructed using the full set of WFs and the optimal feature subset (Table 2). The PLSR model resulted in the highest accuracy for both the training and validation datasets. The validation accuracy of the PLSR model was greater when sensitive features were used (*R*^2^ = 0.51, RMSE = 0.42%, and RRMSE = 7.32%) than when full WFs were used. Since the validation accuracies of the remaining models were low, subsequent regression models were constructed using PLSR.

**Table 2.** Accuracy of 4 models for estimating GPC based on WFs (the full set and the optimal subset). The highest *R*^2^ value and lowest RMSE and RRMSE for each feature set (full and optimal) for the training and validation datasets are shown in bold.

Features	Criteria	BLR		PLSR		RFR		SVMR	
		Training	Validation	Training	Validation	Training	Validation	Training	Validation
Full WFs	*R* ^2^	0.23	0	**0.81**	**0.41**	0.19	0	0.28	0
	RMSE (%)	0.61	0.71	**0.30**	**0.48**	0.63	0.70	0.59	0.73
	RRMSE (%)	11.16	12.33	**5.49**	**8.34**	11.53	12.15	10.80	12.67
Sensitive features	*R* ^2^	0.23	0	**0.86**	**0.51**	0.25	0	0.35	0
	RMSE (%)	0.62	0.72	**0.26**	**0.42**	0.58	0.65	0.50	0.59
	RRMSE (%)	11.35	12.50	**4.73**	**7.32**	10.61	11.28	9.15	10.24

#### Feature extraction and GPC estimation

Table 3 summarizes the accuracy of the PLSR models for estimating GPC using different training sample sizes. For the models using only measured samples, the estimation performance improved with increasing sample size. However, when the simulated data were progressively added to the training data, the estimation accuracy of the model tended to increase initially. For the full WF dataset, the PLSR model achieved the highest accuracy for the validation dataset using 200 simulated samples (*R*^2^ =0.50, RRMSE = 7.27%). For models using sensitive features, 200 simulated samples also produced the highest accuracy (*R*^2^ =0.58, RRMSE = 6.70%). It is noteworthy that as the number of simulated samples increased beyond 200, the accuracy of the models rapidly decreased.

**Table 3.** Estimation accuracy of GPC using the PLSR model based on WFs and with training datasets of different sizes. The highest *R*^2^ value and lowest RRMSE for each feature set (full and optimal) for each of the training and validation datasets are shown in bold. In the column labeled, “Number of training samples,” entries written in the form +*n* indicate 276 measured training samples plus *n* simulated samples.

Number of training samples	Full WFs				Sensitive features			
	Training		Validation		Training		Validation	
	*R* ^2^	RRMSE (%)	*R* ^2^	RRMSE (%)	*R* ^2^	RRMSE (%)	*R* ^2^	RRMSE (%)
50	0.73	6.14	0.05	11.25	0.66	6.81	0.14	10.97
150	**0.86**	4.61	0.40	9.24	0.82	5.15	0.48	8.35
276	0.81	5.49	0.41	8.34	**0.86**	**4.73**	0.51	7.32
+50	0.81	5.51	0.45	7.92	0.83	5.03	0.48	7.60
+100	0.82	5.41	0.43	8.02	0.83	5.03	0.50	7.02
+200	**0.86**	**4.58**	**0.50**	**7.27**	0.85	4.91	**0.58**	**6.70**
+300	0.85	4.81	0.48	7.63	0.70	6.34	0.50	7.39
+500	0.81	5.76	0.32	9.54	0.48	8.54	0.32	9.67
+700	0.71	6.38	0.28	9.60	0.36	9.32	0.09	10.29
+997	0.63	7.89	0.21	10.37	0.12	10.23	0.00	14.20

**Fig. 6.**
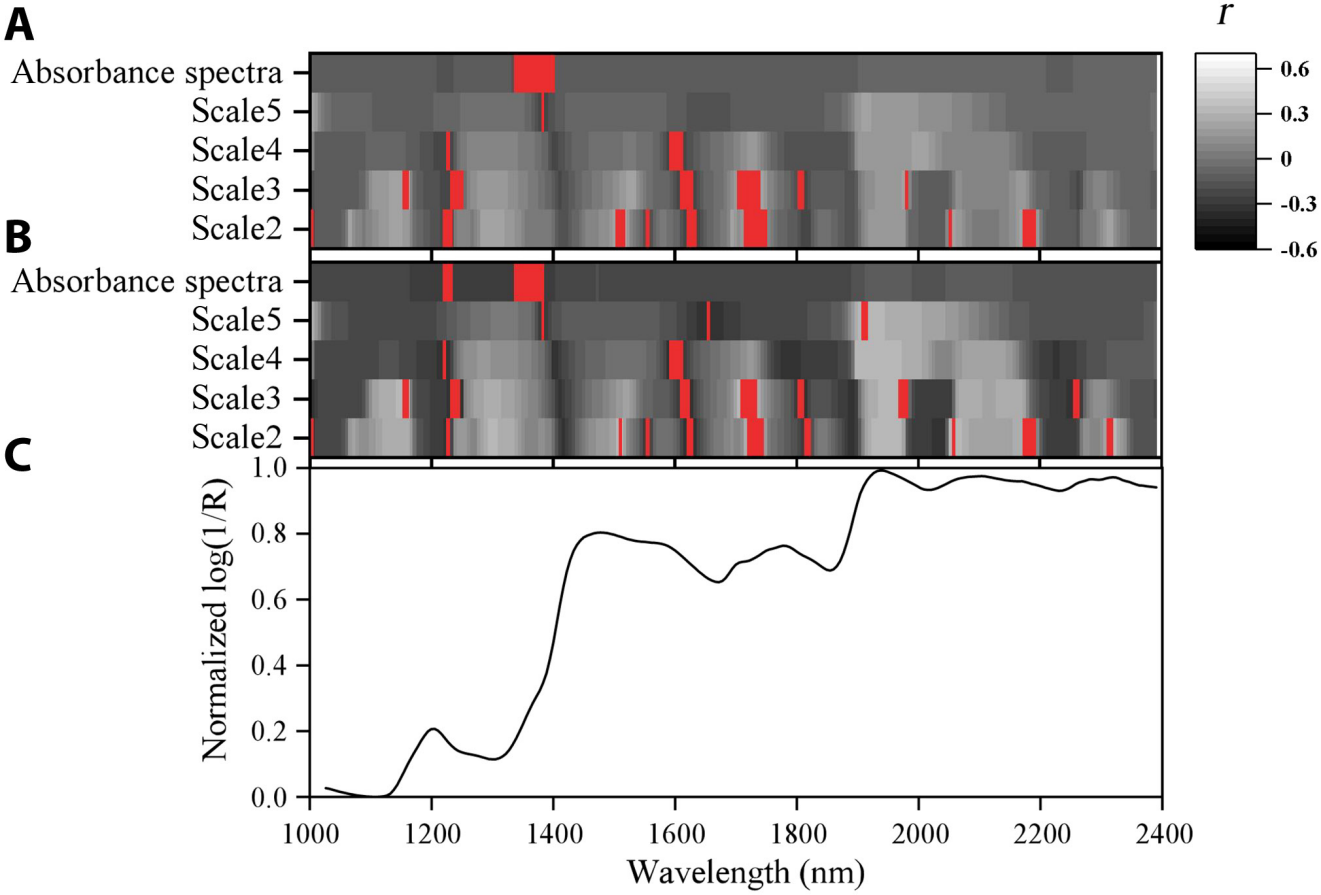
Distribution of GPC-sensitive features based on original and measured data (A), after data augmentation (B), and the average normalized spectral curve (C). Note: Red indicates the top 5% of sensitive features.

Figure 6 shows the distribution of the highest 5% correlation bands followed by recursive feature elimination to GPC in the original data and in the data after augmentation. Across the entire spectral range, the WFs of scale2 and scale3 were generally more highly correlated with the GPC than with the absorbance spectra. The highest correlation between the absorbance spectra and GPC was only 0.26, while the WFs reached a maximum correlation of 0.638. When the scales were large (scale4 and scale5), the correlation of WFs with GPC tended to decrease. After adding the simulated data, the sensitivity of the features to GPC increased, but the highest correlation decreased. The highest correlations before and after data augmentation were 0.638 and 0.609, respectively, corresponding to the WFs WF_1743, 2_ and WF_1738, 2_. The WFs sensitive to GPC for both the real and the combined real and simulated data were dominantly distributed between 1,500 and 1,750 nm, with numerous WFs near 1,600 and 1,700 nm. The addition of the simulated data helped to make the sensitive features more concentrated near the protein absorption bands, while the number of sensitive features in nonprotein absorption regions near 1,250 nm decreased.

### GWAS of measured and estimated GPC

**Fig. 7.**
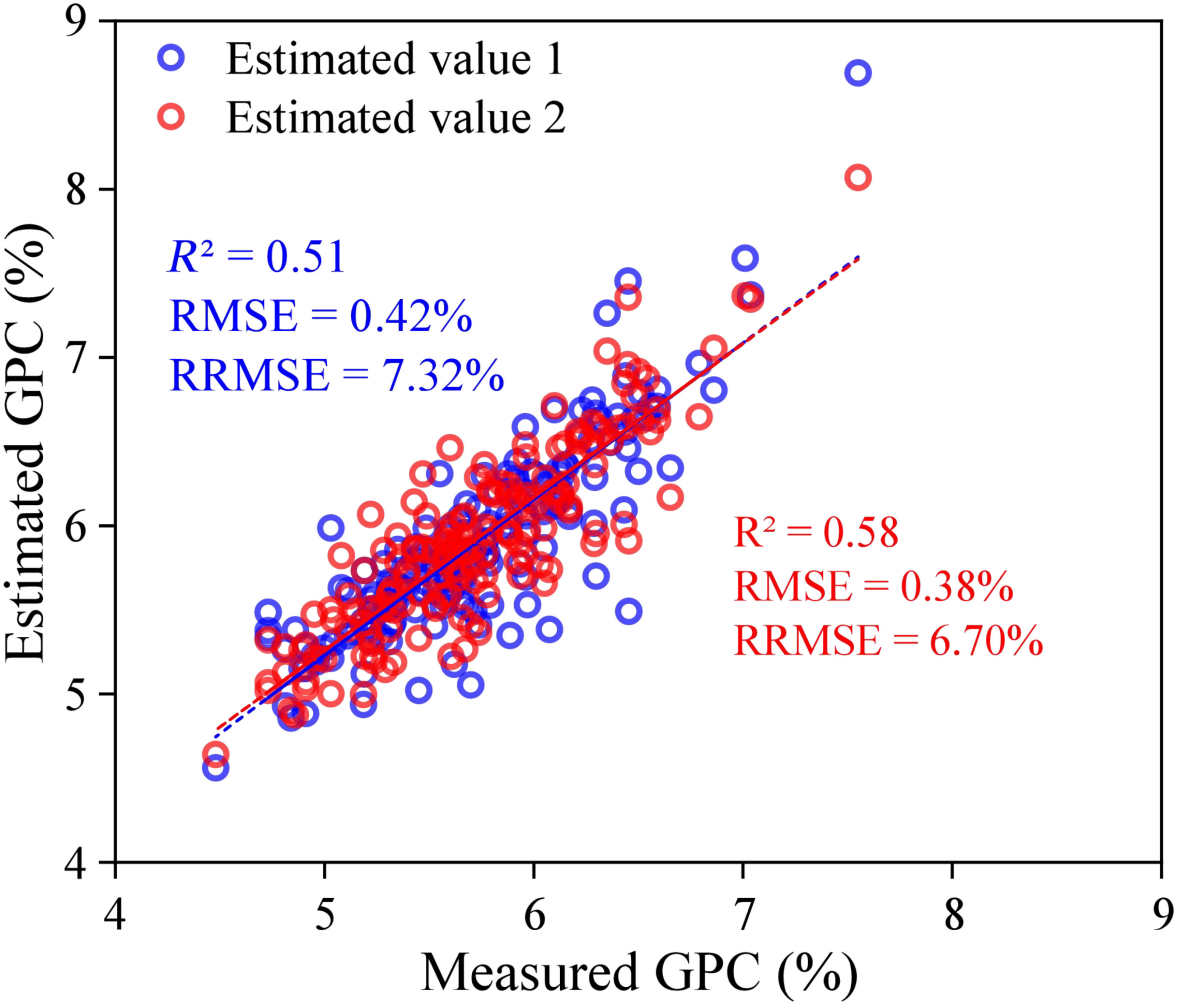
Relationship between the measured and estimated GPC values based on WFs.

GWAS analysis was conducted using the original GPC values, the estimated GPC values inversed by the sensitive features, and the estimated GPC values inversed by the simulated data in this study. The 2 estimated GPC values are expressed as estimated value 1 and estimated value 2. Figure 7 is a scatterplot of the estimated versus the measured values. Compared to the estimated value 1, the estimated value 2 is more concentrated around the 1:1 line and better estimates the outlier points. The estimated values of *R*^2^, RMSE, and RRMSE are 0.58%, 0.38%, and 6.70%, respectively.

**Fig. 8.**
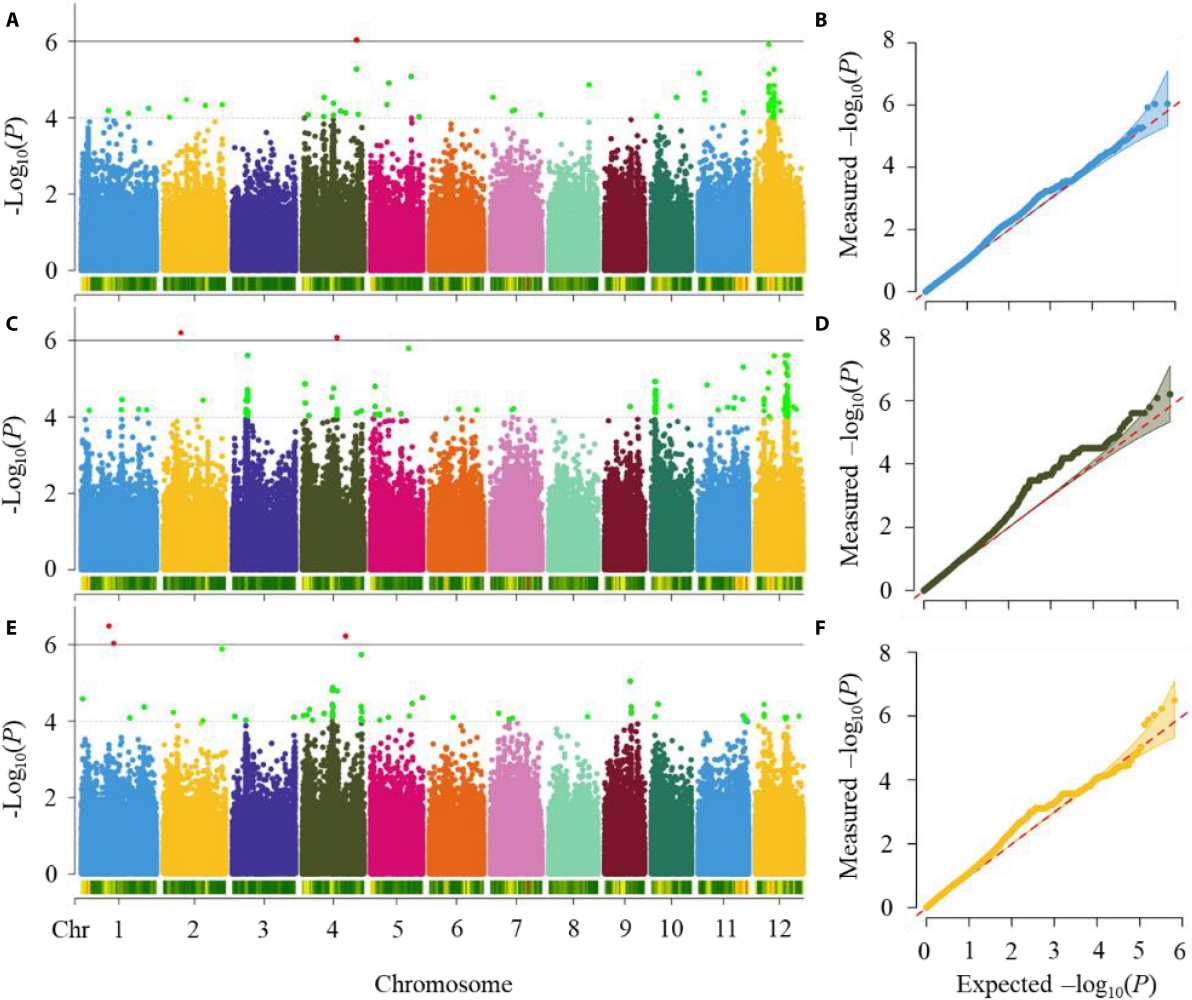
Manhattan (A, C, and E) and quantile–quantile (B, D, and F) plots of measured GPC estimated value 1 and estimated value 2.

Figure 8 shows the Manhattan and quantile–quantile plots of 3 different traits. The detection power of measured and estimated GPC for significant SNPs was not strong, and most SNPs could only reach a significance threshold of log_10_(*P*) = 4. The significant SNPs detected for the 3 traits were scattered across the genome, with only a few regions where a large number of significant SNPs could be detected. GPC, estimated value 1, and estimated value 2 were measured for 4 (SNP4.31180557, SNP7.10830236, SNP12.5076465, and SNP12.7829593), 13 (SNP3.7901940, SNP3.8793654, SNP4.1646356, SNP4.19944999, SNP5.2636506, SNP5.10266233, SNP7.12377473, SNP10.2131826, SNP10.11203648, SNP12.5052003, SNP12.16995867, SNP12.17613781, and SNP12.18719360), and 7 (SNP4.17571584, SNP4.33973841, SNP5.10269557, SNP5.22136770, SNP7.10830048, SNP9.15318049, and SNP12.5052003) lead SNPs.

To compare the similarities of the lead SNPs detected by the different features, a threshold of 100 kb was set. All 3 traits were located at the same locus (SNP12.5076465), and the estimated value 2 overlapped with both the measured GPC (SNP12.5076465 and SNP7.10830236) and the estimated value 1 (SNP12.5076465 and SNP5.10266233) with 2 lead SNPs (Fig. S3). Only SNP4.17571584, detected by estimated value 2, was located in the *OsmtSSB1L* gene, which is related to a grain storage protein. The *OsmtSSB1L* mutant *osmtssb1lcrp* did not cause observable phenotypic changes, but the seeds of the double mutant *ta1* and *osmtssb1lcrp-1* had a thicker dextrin layer than did those of *ta1*. Although the estimated value 1 could detect more lead SNPs, only one locus overlapped with the measured value, and most of them differed from the localization results of the measured GPC. Therefore, the DCGAN-based GPC predictions are effective replacements for the measured values for genetic analysis.

## Discussion

### Feasibility assessment of the DCGAN for generating simulated data

Spectral information has been widely used to construct models for predicting crop physiochemical traits [7,40,44]. However, it is difficult to obtain a large number of high-quality samples, which may result in the model being unable to learn the correct distribution of the samples, thereby affecting the generalizability of the model [45]. However, data augmentation has the potential to play a role in regularization, preventing overfitting, and improving model performance [28]. Zhang et al. [32] reported that DCGANs could be used to generate spectral data and corresponding chemical values, and the addition of generated data improved the performance of PLSR models, which was consistent with the results of this study.

To achieve highly accurate GPC estimates, a DCGAN model for data augmentation was constructed. The data used to train the model were one-dimensional curves comprising “spectral data + measured traits,” which was similar to the approach of Zhang et al. [32]. However, in this study, GPC was inserted into the protein-sensitive band after 1,510 nm, while Zhang et al. [32] placed the seed oil content at the end of the curve. This difference can be ascribed to the fact that Zhang [46] acquired spectra of seeds of individual maize varieties, a dataset with less variability and less noise than our dataset has. In our study, the spectral data represent a large number of rice varieties, with large differences in chemical composition and internal structure among the varieties. Inserting the GPC data after 1,510 nm was logical, given that the variation trend of the band near 1,510 nm was similar to that of GPC. This facilitated convergence in the model, and in addition, the influence of noise from the edge of the band was avoided.

For this dataset, the simulated spectra generated by the DCGAN model at epoch 8000 were the most similar to the measured spectra. For the simulated GPC, all statistics were very close except for the minimum value, which was smaller than the measured value (Table S2), and the interquartile range of the simulated GPC was slightly biased toward higher protein content (Fig. 5). According to the evaluation criteria of Sun et al. [47], this indicates that the generated data distribution was reasonable and that the diversity of the simulated data was greater than that of the measured data, satisfying the demand of regression models for high variability in training data. Furthermore, the generated data adequately represented the distribution of the measured data and made up for the uneven distribution of the original GPC. The increase in the number of samples provided by the simulated data improved the model estimation accuracy (at least up to a point) without losing the features obtained before data augmentation.

### Comparison of sensitive features between simulated and measured datasets

The absorbance spectral bands that were identified before and after data augmentation as sensitive to GPC were relatively consistently concentrated at approximately 1,350 nm. However, the correlation between the original absorbance spectra and GPC was weak, and even after data augmentation, the maximum correlation was only 0.26 (Fig. 6). These low correlations may be explained by the translucent appearance of the rice grains and the weak reflection signal in the SWIR range [8]. In addition, it is noteworthy that a focus on the correlation of individual bands with GPC ignores the broader spectral response across multiple wavelengths.

CWT can capture subtle feature changes in spectral curves at small scales and overall changes in curves at large scales, thereby enhancing the sensitivity of WFs to target traits [21,46,48]. The wavelet transform increased the correlation to GPC from a maximum of approximately 0.25 to approximately 0.6 after data augmentation. Both low-scale (scales 2 and 3) and high-scale (scales 4 and 5) wavelet decomposition exhibited common sensitive features near 1,250, 1,600, and 1,700 nm, which was consistent with the fact that CWT analysis is based on spectral data rather than the magnitude of reflectance [25].

The WFs found to be sensitive to GPC before and after data augmentation were relatively consistent, were mainly distributed at approximately 1,500 to 1,750 nm, were stable, and were close to the protein absorption bands at 1,510 and 1,690 nm [19]. This provides additional evidence that the simulated data had indeed learned the true distribution of the original data and that the addition of simulated data did not bias the correlation between the original features and GPC. The increase in the amount of simulated data helped to concentrate the sensitive features near the protein absorption bands. This is mainly because the addition of simulated data compensates for the lack of data for high GPC samples, making the training set more representative of the wide range of GPC values and distribution of the GPC data. Thus, the augmented dataset detected more sensitive features near the GPC absorption bands and eliminated some features that may not be sensitive diagnostic features. The final independent verification results also indicated that the model constructed with the selected sensitive features after adding the simulated data had the highest accuracy (Table 3).

### Effect of sample size on the estimation accuracy of regression models

Increasing the size of the training set improved the model performance at least initially, and the estimation accuracy reached its highest value with 200 simulated samples. This indicates that the process for generating simulated data learned some of the distribution characteristics from the original data, and adding simulated data made the GPC distribution closer to the measured distribution.

However, the benefit of increasing the data volume for improving model performance was limited. When the number of samples exceeded a certain threshold, the model performance rapidly decreased (Fig. S4). This suggests that after some point, increasing the number of samples merely adds noise [32]. Compared with PLSR models based on the full range of WFs, PLSR models based on sensitive features often achieved better results for the same dataset. However, when the number of samples was small or there were too many simulated samples, the model performance notably decreased. This may be because models based on a few features are more susceptible to interference from noise.

In summary, using a DCGAN to generate simulated datasets improved the estimation performance, and this improvement was greater for PLSR models based on sensitive features. However, when the simulated data volume was too large, the estimation accuracy of the model rapidly decreased. Therefore, the impact of factors such as the type of model, estimation model, simulation data volume, and feature selection method on the estimation results should be comprehensively considered when building a high-precision estimation model when using a DCGAN to expand the dataset.

### The feasibility of gene location through GWAS analysis with measured and estimated GPC values

In this study, GWAS results of measured GPC and estimated GPC revealed that they both had only a weak ability to detect significant loci and lead SNPs. This may be the consequence of a lack of replication in the 2 years of field trials and the greater influence of the environment on GPC in the second year, which resulted in, on average, high GPC levels. Through GWAS, more lead SNPs could be detected in the estimated values than in the measured values, and only the estimated value of 2 could be used to detect the grain storage protein-related gene *OsmtSSB1L*. This may be because the GPC estimates were derived from the integration of spectral information across multiple bands, and spectral reflectance represents a comprehensive response to changes in specific chemical bonds (N–H bonds) in a substance. Therefore, the model incorporated richer seed protein-related information into the GPC estimates [14,15].

Both estimated value 1 and estimated value 2 could detect the same lead SNPs as the measured value (Fig. 7). This confirmed that the hyperspectral-based GPC estimates had genetic information similar to that of the measured values. Although the accuracies of estimated value 1 and estimated value 2 were similar, there was a significant difference in the GWAS results between them. More lead SNPs could be detected from the GPC estimates based on the original data, and most of them were inconsistent with the localization results of the measured GPC. Some significant loci detected by measured GPC were not detected by estimated values, and the lead SNPs detected by measured values were less than those detected by estimated values. This was because the estimated values were not exactly equivalent to the measured values. The highest correlation between the estimated and measured values in the study was 0.86. High-accuracy GWAS analysis is required for phenotype analysis, and even subtle differences may affect the results [43]. A further complexity was that some loci were false positives. The best linear unbiased predictions (BLUPs) or best linear unbiased estimates (BLUEs) should be calculated to address environmental effects [49]. However, the same varieties collected in both years were limited due to severe lodging and disease; hence, BLUP/BLUP experiments were not performed in this study. Due to the limited number of the same varieties and the significant differences in GPC between the 2 years, there was a high probability that the detected loci were false-positive loci generated by noise [50]. In future studies, the phenotypes of more rice varieties should be collected from multiple years and ecological sites to assess the methodology of this study.

## Conclusion

This study focused on solving the problem of limited numbers of training samples for GPC estimation, which leads to low estimation accuracy and poor generalization ability. DCGAN was employed to generate grain spectra and GPC-simulated data for data augmentation. A PLSR model for estimating GPC was constructed using WFs found to be sensitive to GPC. By comparing the GWAS results, the feasibility of using GPC estimated values instead of measured values for genetic dissection was clarified. The main conclusions are as follows:

1. DCGANs can generate spectral and simulated GPC data to solve the problem of limited measured data. Compared to the measured data, the simulated GPC distribution range after 8,000 epochs was wider, and the proportion of samples with high protein content (>5.2%) was greater.

2. Data augmentation can improve the accuracy of GPC estimation models. When the number of simulated samples reached 200, the PLSR model based on WFs exhibited the best performance (*R*^2^ = 0.58 and RRMSE = 6.70%), and the sensitive features were near the protein absorption bands at 1,510 and 1,690 nm.

3. GPC data estimated using DCGANs have the ability to replace measured values for genetic analysis. The estimated values based on the DCGAN could identify 2 lead SNPs that were identical to the measured values (SNP7.10830236 and SNP12.5076465). The estimated values revealed an additional 5 loci. Among them, SNP4.17571584 was able to detect the *OsmtSSB1L* gene, which is related to a grain storage protein.

## Data Availability

The datasets, source code, and other supporting data are available upon request.
