# Supplementary material for: Grain Protein Content Phenotyping in Rice via Hyperspectral Imaging Technology and a Genome-Wide Association Study
Source: Plant Phenomics. 2024 Jul 8;6:0200. doi: 10.34133/plantphenomics.0200 (PMC11227985; doi:10.34133/plantphenomics.0200)
Supplement: Supplementary 1 — Figs. S1 to S4 Tables S1 and S2 [file plantphenomics.0200.f1.docx]

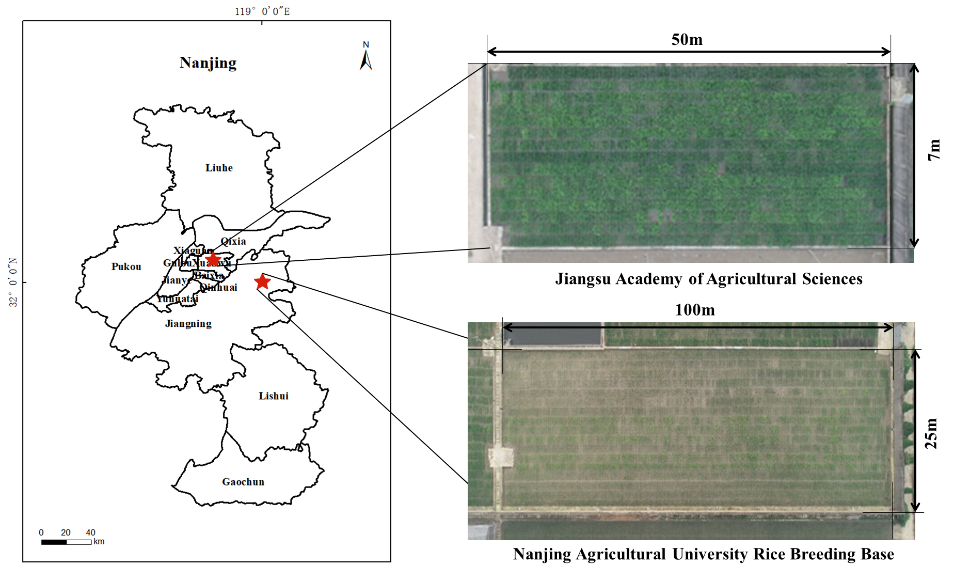


F_IGURE_ S1: Geographic location of the experimental sites and view of rice field.


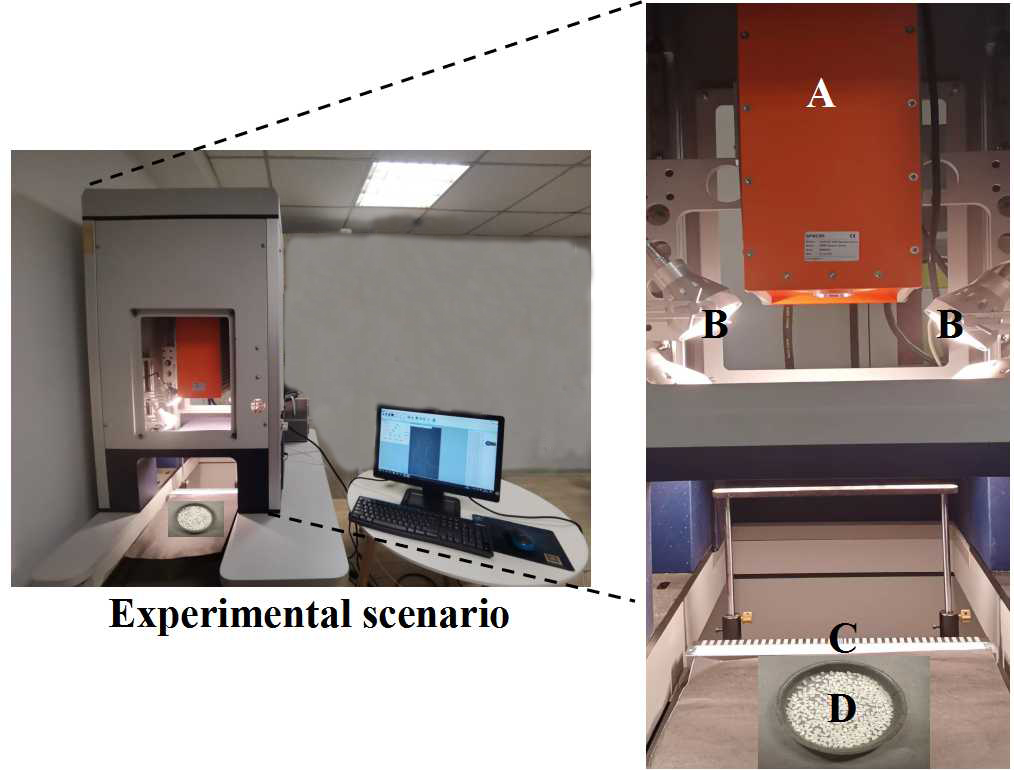


F_IGURE_ S2: The push-broom SWIR system uesed in this study.

A: N25E hyperspectral imager; B: halogen lamp; C: calibration board; D: sample


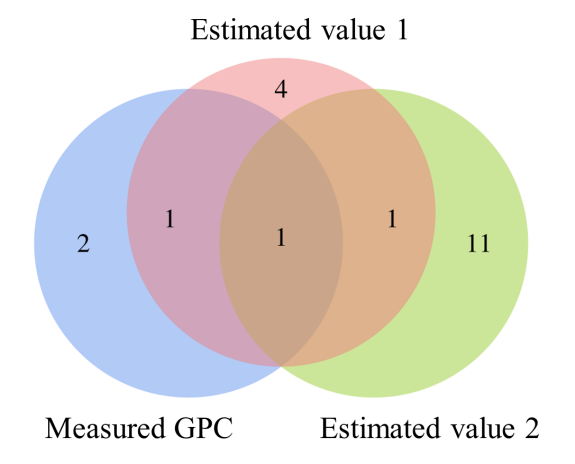


F_IGURE_ S3: Venn diagram of lead SNPs detected by three traits.


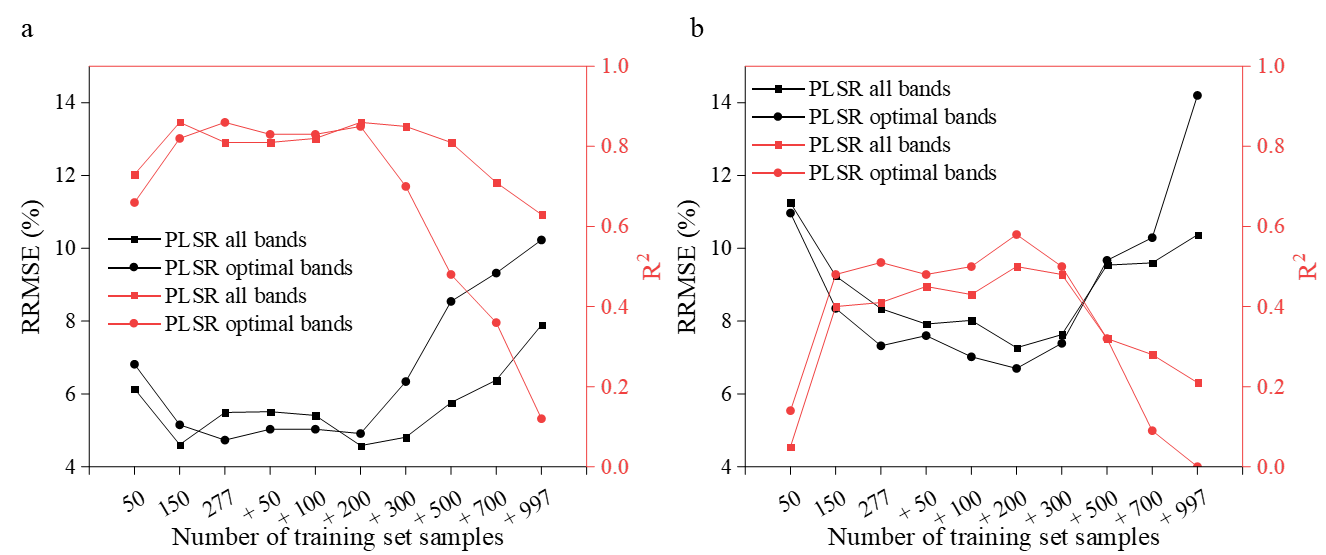


F_IGURE_ S4: Training set accuracy (a) and validation set accuracy (b) with different numbers of training samples.

T_ABLE_ S1: Number of varieties planted and harvested in the two years of trials.

| Experiment | Site | Year | Number of  varieties planted | Number of  varieties harvested |
| --- | --- | --- | --- | --- |
| 1 | Jiangsu Academy of Agricultural Sciences | 2020 | 230 | 126 |
|  |  | 2021 | 230 | 112 |
| 2 | Nanjing Agricultural University Rice Breeding Base | 2020 | 190 | 85 |
|  |  | 2021 | 320 | 191 |
| All | All | 2020 & 2021 | 970 | 514 |

T_ABLE_ S2: Summary statistics of the GPC measured and simulated training data, and measured validation data.

| Dataset | | Number of samples | GPC (%) | | | | |
| --- | --- | --- | --- | --- | --- | --- | --- |
| Training | Validation |  | Minimum | Maximum | Mean | Standard deviation | CV |
| Measured |  | 276 | 4.17 | 7.44 | 5.54 | 0.71 | 0.13 |
| Simulated |  | 997 | 3.99 | 7.42 | 5.53 | 0.70 | 0.13 |
|  | Measured | 238 | 4.48 | 7.55 | 5.72 | 0.60 | 0.10 |
